# Two views of the same stimulus

**DOI:** 10.7554/eLife.30191

**Published:** 2017-08-14

**Authors:** Wayne A Johnson

**Affiliations:** 1 Department of Molecular Physiology and BiophysicsUniversity of IowaIowa CityUnited States

**Keywords:** adhesion GPCR, mechanotransduction, metabotropic signalling, sensory physiology, dCIRL, latrophilin, *D. melanogaster*

## Abstract

Signals from two different membrane proteins are combined to modulate how strongly sensory neurons respond to mechanical force.

**Related research article** Scholz N, Guan C, Nieberler M, Grotemeyer A, Maiellaro I, Gao S, Beck S, Pawlak M, Sauer M, Asan E, Rothemund S, Winkler J, Prömel S, Nagel G, Langenhan T, Kittel RJ. 2017. Mechano-dependent signaling by Latrophilin/CIRL quenches cAMP in proprioceptive neurons. *eLife* **6**:e28360. doi: 10.7554/eLife.28360

The ability of sensory neurons to detect and respond to mechanical force using a process known as mechanotransduction allows us to interpret and navigate the physical world around us. Although a number of different proteins have been linked to mechanotransduction, how they interact with each other or with other signaling pathways in a single sensory neuron is still not well understood.

The study of mechanotransduction has focused primarily upon ion channels that span cell membranes and open in response to mechanical perturbation. In most cases, the channel protein itself is the mechanosensor, responding either to changes in the physical properties of the cell membrane or the tension in a molecular anchor within the cell (Ranade et al., 2015). This promotes the false impression that each type of channel operates independently. However, individual sensory neurons normally express a variety of ion channels and other proteins that allow them to respond to many different signals, such as touch, chemicals, and both hot and cold temperatures (Geffeney and Goodman, 2012; Abraira and Ginty, 2013).

Recent studies have begun to unravel the complex relationship between mechanosensory channels and their environment inside a single neuron. For example, some channels have been shown to interact with members of a superfamily of membrane proteins called the G-protein coupled receptors (GPCRs). The binding of an external signal molecule (ligand) to a GPCR stimulates signaling pathways inside the cell that are involved in a large variety of processes. Furthermore, some of the components in these pathways can interact with mechanosensory channels in response to pain or inflammation (Geppetti et al., 2015; Veldhuis et al., 2015).

All GPCRs contain a seven transmembrane domain embedded within the cell membrane, along with one or more domains inside the cell that trigger the downstream signaling pathways. Members of a subgroup known as the adhesion GPCRs also contain an unusual extracellular domain (ECD) that is thought to interact with components of the extracellular matrix, a scaffold-like structure that surrounds cells to provide structural support (Langenhan et al., 2016)

The ECD is linked to the transmembrane domains by another domain that allows some adhesion GPCRs to cut themselves into two pieces. It has been assumed that this “autoproteolysis” step, which splits the ECD away from the rest of the protein, is essential to activate adhesion GPCRs. Recent studies suggest that some adhesion GPCRs may detect mechanosensory information through the ECD when it is tethered to the extracellular matrix (Petersen et al., 2015; Scholz et al., 2015).

In 2015, a team of researchers led by Robert Kittel and Tobias Langenhan at the University of Würzburg reported that an adhesion GPCR called dCIRL may influence the activity of the NOMPC mechanosensory channel in the chordotonal organ of fruit fly larvae (Scholz et al., 2015). However, it was not clear whether the two proteins directly interact with each other. Now, in eLife, Kittel, Langenhan and co-workers – including Nicole Scholz as first author – report that dCIRL may modulate the activity of NOMPC by stimulating signaling pathways inside the neuron (Scholz et al., 2017).

The chordotonal organ plays crucial roles in a range of mechanosensory processes in fruit fly larvae, and Scholz et al. found that dCIRL (also known as latrophillin) and NOMPC co-localize to the same structures within the neurons in this organ. Mechanical stimuli trigger weaker responses in mutant larvae that are unable to produce dCIRL than they do in normal larvae. In addition, changing the length of the ECD modified the response of dCIRL to mechanical stimuli consistent with an essential role for the ECD in transducing the signal. Together, these results suggest that dCIRL interacts with unidentified ligands outside of the cell to modulate the activity of NOMPC and adjust how strongly a neuron responds to a mechanical stimulus.

Unlike the adhesion GPCRs studied in other animals, dCIRL does not require autoproteolysis to be correctly localized or activated in neurons. Moreover, dCIRL also appears to use different signaling pathways because it decreases the level of a signal molecule called cAMP in cells, whereas the adhesion GPCRs in other animals have the opposite effect (Müller et al., 2015). By using different signaling pathways, the various members of this GPCR subgroup may play different roles in different cell types or species.

The findings of Scholz et al. provide a potential process by which multiple signaling events (such as the complementary mechanical inputs from NOMPC and dCIRL) can interact within a single sensory neuron to precisely modulate the neuron's response to a mechanical perturbation (Figure 1). The diversity of the adhesion GPCR proteins across different cell types and species supports the hypothesis that the way adhesion GPCRs modulate mechanotransduction may also depend upon the type of mechanical signal they receive.

**Figure 1.**
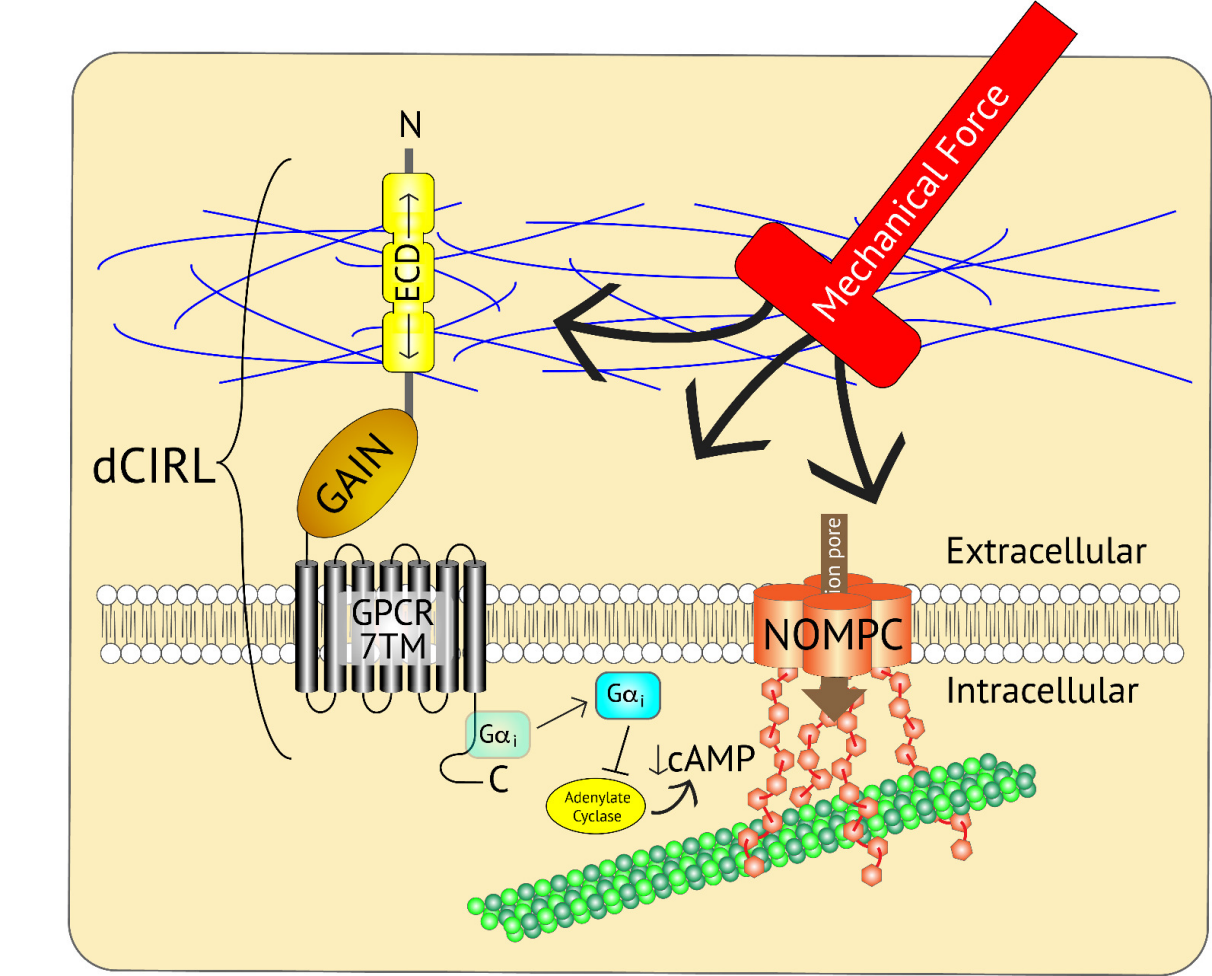
Adjusting the mechanosensory response of sensory neurons. The ion channel NOMPC (orange) is a transmembrane protein that is anchored to the neuron’s cytoskeleton (green circles), allowing it to detect mechanical force. This stimulates the pore of the channel (brown arrow) to open, leading to the generation of an electrical signal within the neuron. At the same time, the extracellular domain (ECD) of another transmembrane protein, the adhesion GPCR dCIRL, detects the mechanical force differently as a result of being anchored to the extracellular matrix (blue strings). Activation of dCIRL decreases the level of cAMP in the cell, possibly due to the activation of a G protein (Gαi) that inhibits the enzyme that makes cAMP (known as adenylate cyclase). Unidentified signaling components downstream of cAMP alter NOMPC activity to modulate the strength of the overall response. In this manner, both extracellular and intracellular views of the same stimulus are combined to precisely adjust the neuronal response. GAIN, GPCR autoproteolytic domain. 7TM: seven transmembrane domain.
